# Long-term follow-up of torpedo maculopathy: a case series and mini-review

**DOI:** 10.1186/s12886-023-03254-z

**Published:** 2024-01-03

**Authors:** Richard C. Trevino, William H. Ridder, Anupam Laul, James Hill

**Affiliations:** 1 School of Optometry, Indiana University, 800 Atwater Ave Bloomington, Bloomington, IN 47405 USA; 2 Southern California College of Optometry, Marshall B. Ketchum University, Fullerton, CA USA; 3 College of Optometry, State University of New York, New York, NY USA; 4 College of Medicine, Medical University of South Carolina, Charleston, SC USA

**Keywords:** Torpedo maculopathy, Gardner syndrome, Multimodal imaging, Retinal diseases, congenital, Retinal diseases, diagnostic imaging, Case report

## Abstract

**Background:**

Torpedo maculopathy (TM) is a rare, congenital condition characterized by an oval-shaped, chorioretinal lesion in the temporal macula of unknown etiology. To our knowledge, the longest reported follow-up of TM is 5 years. Herein we report 10 years of follow-up on two patients with TM to further characterize the long-term natural history of the condition.

**Case reports:**

Two patients with torpedo maculopathy were examined at baseline and then again at 5 years and 10 years from baseline. Eyes were evaluated using color fundus photography, automated perimetry, fundus autofluorescence and spectral domain optical coherence tomography. Visual function of both patients remained stable throughout the observation period. In case 1, there was no evidence of change in lesion morphology over the 10 year observation period. Case 2 showed progression of cystic degeneration of the neurosensory retina within the torpedo lesion. Case 1 reported a history of supernumerary teeth and underwent gene sequence with deletion/duplication analyses of the APC gene but no clinically significant variants were detected.

**Conclusions:**

Our findings support the position that TM is a nonprogressive condition with long-term stability of visual function. Genetic analysis of case 1 failed to detect any association with Gardner syndrome.

## Background

Torpedo maculopathy (TM) is a rare, congenital condition characterized by an oval-shaped, chorioretinal lesion in the temporal macula of unknown etiology [1]. It is believed to represent a developmental defect of the eye, but theories vary as to whether it is primarily a defect in the development of the retinal nerve fiber layer [2], retinal pigment epithelium [3] or choriocapillaris [4].

The clinical features of TM include a unilateral, solitary, hypopigmented lesion in the temporal macula having a fusiform (“torpedo”) shape with its long-axis oriented horizontally and its nasal edge pointed toward the fovea [1]. However, many variations of this typical presentation have been reported [5–9]. Common variants include presence of outer retinal cavitation, choroidal excavation, and hyperpigmentation within the torpedo lesion. Less common variants include bilaterality, multiplicity, and atypical fundus location. The diagnosis of torpedo maculopathy is clinical and based upon recognizing the distinctive features of the condition.

There is some question as to whether the variability in clinical features of TM represents differences in phenotype or different stages of a progressive disorder [8, 10–13]. To date, the longest published follow-up of TM is 5 years. Most reports with follow-up have found torpedo lesions to be stationary, but two case reports each with 5 years of follow-up found morphologic changes of the lesion over time [8, 11].

The purpose of this paper is to present the 10 year follow-up findings of two patients with TM. To our knowledge, this represents the longest reported follow-up of any patient with TM.

## Case 1

**Fig. 1:**
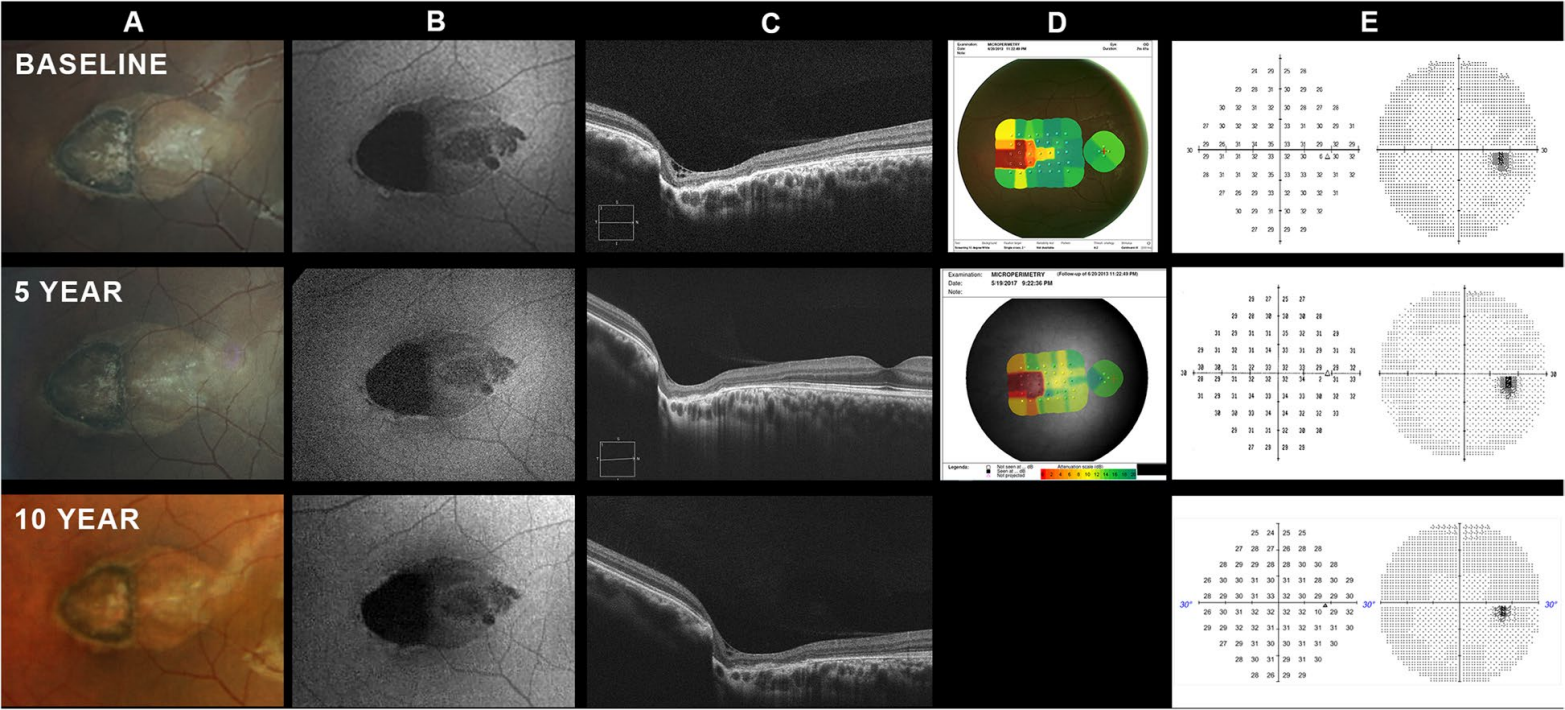
Case 1. **A **Color fundus photography demonstrates a horizontally oval, hypopigmented, torpedo-shaped lesion in the temporal macula. Serial photography documents apparent stability of the torpedo lesion over the 10 year observation period. **B **Fundus autofluorescence demonstrates typical features of TM, including hypoautoflurescence of the lesion interior and hyperautofluorescence of the lesion margins. There is no apparent change in the signal pattern over the observation period. **C **Spectral domain optical coherence tomography (OCT). Each image represents a horizontal B-scan through approximately the same location in the middle of the torpedo lesion. Because the examinations were performed on different instruments at each time period, the scans are not of the identical fundus location. Nonetheless, they are as close to the same fundus location as possible. Comparison of the images suggests that the lesion has remained relatively stable over the observation period. **D **Microperimetry was only performed at baseline and the 5 year follow-up visit. No change in the size or depth of the scotoma produced by the deeply excavated temporal aspect of the torpedo lesion was detected. **E **Standard automated perimetry was normal at each visit

This patient initially presented to the Rosenberg School of Optometry eye clinic in 2012. Her baseline findings have been previously reported [14]. In summary, she was a healthy 25-year-old South Asian female who was discovered to have a torpedo lesion in her left eye during routine examination. Her best corrected visual acuity was 20/15 in each eye. The fundus of each eye was normal with the exception of a torpedo-shaped, excavated lesion temporal to the fovea in the right eye (Fig. 1A). Fundus autofluorescence revealed patchy regions of hyper-autofluorescence along the margins of the lesion. The temporal half of the lesion was densely hypo-autofluorescent with patchy regions of hypo-autofluorescence within the nasal portion of the lesion (Fig. 1B). Optical coherence tomography (OCT) revealed that the temporal aspect of the lesion was steeply excavated with severe disorganization of the outer retinal layers and loss of inner choroidal layers (Fig. 1C). The lesion became less excavated nasally with gradual recovery of normal retinal and choroidal structure at its nasal margin. Standard automated perimetry using the 30 − 2 test pattern was normal for both eyes, but a scotoma corresponding to the temporal half of the lesion could be demonstrated using microperimetry (Fig. 1D-E).

The patient returned for follow-up in 2017 (Fig. 1, YEAR 5) and again in 2023 (Fig. 1, YEAR 10). No changes in visual function or lesion morphology were noted at either follow-up visit. Because the patient had relocated, her 2023 exam was conducted at the SUNY College of Optometry eye clinic. At that time her ocular and medical history remained unremarkable. However, upon direct questioning the patient reported a history of supernumerary teeth. She denied a history of cranial osteomas, epidermal cysts, intestinal polyposis, or brain and other tumors. Given the possible association between TM and Gardner syndrome [15], the patient underwent gene sequence and deletion/duplication analyses of the APC gene. No clinically significant variants were detected. The patient also underwent colonoscopy with no abnormalities found. The patient’s blood relatives were not available for examination, but the patient informed us that her immediate blood relatives underwent eye examinations and no retinal lesions were uncovered. The patient was advised to self-monitor her vision with an Amsler grid.

## Case 2

**Fig. 2:**
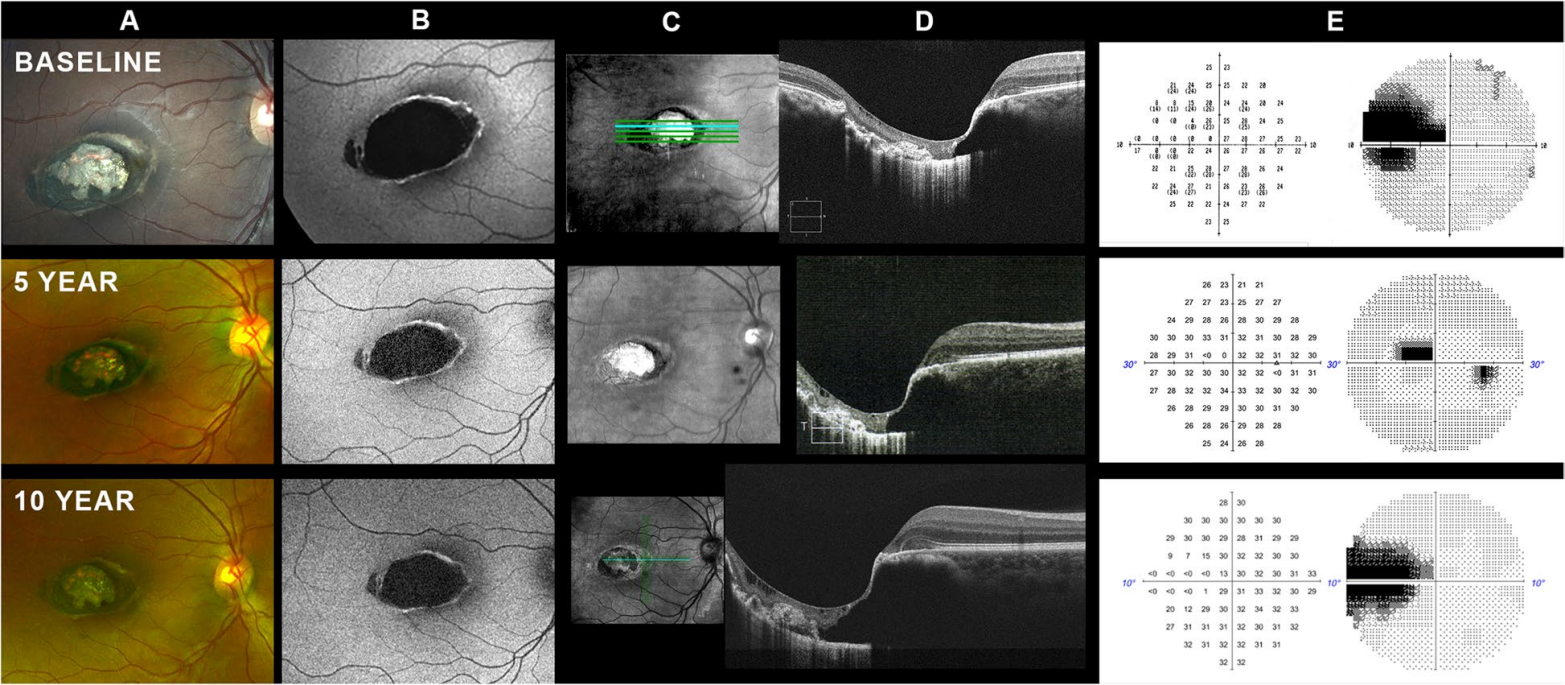
Case 2. **A **Color fundus photography demonstrate a horizontally oval, hyperpigmented, torpedo-shaped lesion containing a central pseudolacuna in the right eye. A small satellite lesion is noted adjacent to the temporal margin of the lesion. Serial photography documents apparent stability of the lesion over the 10 year observation period. **B **Fundus autofluorescence demonstrates typical features of TM, including hypoautoflurescence of the lesion interior and hyperautofluorescence of the lesion margins. The lesion is interrupted near the temporal margin by a tongue of tissue with normal autofluorescence signal that splits the lesion into two parts. This corresponds to the observed satellite lesion at the temporal edge of the torpedo lesion. There is no apparent change in the signal pattern over the observation period. **C **Scanning laser ophthalmoscopy images with location of corresponding B-scan highlighted in the baseline and 10-year follow-up exam. **D **Spectral domain optical coherence tomography (OCT). Each image represents a horizontal B-scan through approximately the same location in the middle of the torpedo lesion. Because the examinations were performed on different instruments at each time period, the scans are not of the identical fundus location. Nonetheless, they are as close to the same fundus location as possible. At the 10-year follow-up visit some progression of the inner retinal degeneration is seen. A few small cystic spaces are seen under the inner limiting membrane at baseline, and they appear larger and more numerous at the 10-year visit. While some retinal layers can be identified thoughout most of the lesion in the baseline scan, the 10-year scan reveals an almost complete loss of retinal organization within the torpedo lesion (**E**)Standard automated perimetry reveals a dense scotoma nasal to fixation that corresponds to the torpedo lesion in the temporal macula

This patient initially presented to the Rosenberg School of Optometry eye clinic in 2012. Her baseline findings have been previously reported [14]. In summary, she was a 22 year old African American female who presented for routine eye examination. She reported that the vision of her right eye has always been worse than her left eye for unknown cause. She has a medical history of lupus erythematosus and anemia. Her medication list consisted of hydroxychloroquine, prednisone, methotrexate, metronidazole, and oral contraceptives. Her best-corrected visual acuities were 20/50 and 20/20 in the right and left eyes, respectively. The fundus of each eye was normal with the exception of a torpedo-shaped, excavated lesion involving the macula of the right eye (Fig. 2A). The bulk of the lesion was situated in the temporal macula, but its nasal edge involved the fovea, resulting in the loss of visual acuity. On fundus autofluorescence imaging the lesion was hypo-autofluorescent with a narrow ring of hyper-autofluorescence extending around the margins of the lesion except temporally, where the boundary of the lesion appears less discrete (Fig. 2B). OCT revealed steep excavation at the nasal edge of the lesion with a tapered upward sloping margin temporally (Fig. 2C-D). Standard automated perimetry using the 10 − 2 and 30 − 2 test patterns revealed a dense scotoma nasal to fixation (Fig. 2E).

The patient returned for follow-up in 2017 (Fig. 2, YEAR 5) and again in 2023 (Fig. 2, YEAR 10). Because the patient had relocated, her 2017 examination was conducted at the Southern California College of Optometry and her 2023 exam was conducted at the Medical College of South Carolina eye clinic. No changes in visual function were noted at either follow-up visit. Compared to her 2012 examination, OCT performed in 2023 revealed evidence of cystic degeneration of the neurosensory retina within the torpedo lesion. At the time of her 2023 exam she reported that she had discontinued Plaquenil in 2018 and her lupus was well controlled using belimumab. She denied a personal or family history of supernumerary teeth, cranial osteomas, epidermal cysts, intestinal polyposis, or brain and other tumors. The patient declined genetic testing of the APC gene. The patient has not undergone colonoscopy and her blood relatives were not available for examination. The patient was advised to self-monitor her vision with an Amsler grid.

## Discussion

TM is likely a congenital lesion that results from a developmental defect of the eye. The youngest reported case is from an otherwise healthy and normal 6-month old child [16]. Yet, questions remain regarding the stability of the condition. Wong and colleagues proposed a TM classification scheme based upon lesion morphology [13]. They found that among the 5 patients in their case series and the 9 previously reported patients with OCT findings, patients that had TM associated with outer retinal cavitation (so-called Type 2 lesions) were on average older than patients having TM lesions without outer retinal cavitation (Type 1 lesions) − 17 years of age versus 39 years of age, respectively. They postulated that this is evidence of lesion progression, with outer retinal cavitation appearing as a later, more advanced form of the condition. However, Shirley and associates have reported on a case series of 7 children under 16 years of age with TM and OCT findings seen at their institution and found that patients with outer retinal cavitation were not on average older than children without outer retinal cavitation (7 and 8 years of age, respectively) [12].

Relatively few longitudinal studies of TM have been published, so the question of whether TM is a stable or progressive condition remains unresolved. Our review of the literature revealed 24 cases (including the current report) with greater than 1 year of follow-up (Table 1). Four of these 24 cases (17%) demonstrated some change in the torpedo lesion over the observation period. All four of the patients with documented change had 5 or more years of follow-up. Among all 7 cases with 5 or more years of follow-up, 57% (4 of 7 cases) demonstrated some change in lesion morphology. Reported changes consist of lesion enlargement and degeneration of retinal tissue within the torpedo lesion. Of the two cases presented herein, one patient had OCT evidence of degeneration of retinal tissue over the 10 year observation period while no changes were detected in the other patient. Baseline OCT scan of our Case 2 revealed a small number of hyporeflective cavities within the inner retina located predominantly in the temporal aspect of the torpedo lesion. The cystoid changes were poorly visualized at the 5 year exam but at the 10 year exam they had clearly become larger and more numerous. While fluorescein angiography was not performed, there is no OCT evidence of an exudative process, hence these lesions are likely of non-vasogenic origin, possibly secondary to mechanical/tractional stress induced by the excavation [17]. Of the 4 reported cases of lesion progression none has suffered any impairment of visual acuity as a consequence of lesion progression.

**Table 1:** Reported cases of torpedo maculopathy with greater than 1 year of follow-up

AUTHOR^a^	YEAR	SEX	AGE^b^	F/U	FINDINGS
Current study		F	25	10 Y	No change
Current study		F	22	10 Y	Retinal degeneration
Sanabria-1 [11]	2008	F	12	5 Y	Retinal thinning
Sanabria-2 [11]	2008	F	13	5 Y	Enlargement
Hansen [33]	2016	M	12	5 Y	No change
Rohl [8]	2016	M	10	5 Y	Enlargement and ovalization
Giannakaki-1 [19]	2019	F	8	5 Y	Stable VA
Pian-1 [34]	2003	M	59	4 Y	No change
Golchet-1 [35]	2010	F	33	4 Y	No change
Dutra [36]	2013	F	5	4 Y	No change
Pian-2 [34]	2003	M	63	3 Y	No change
Schuerch-1 [37]	2015	F	3	3 Y	No change
de Manuel-1 [38]	2016	M	4	3 Y	No change
Suarez [39]	2017	F	4	3 Y	No change
Ehrenberg-2 [40]	2023	F	2	3 Y	No change
Ehrenberg-5 [40]	2023	F	7	3 Y	No change
Ehrenberg-6 [40]	2023	M	4	3 Y	No change
Ehrenberg-7 [40]	2023	M	4	3 Y	No change
Ghezzaz [22]	2023	F	74	2.5 Y	CNV Stable
Pian-3 [34]	2003	M	63	2 Y	No change
Giannakaki-2 [19]	2019	F	13	2 Y	Stable VA
Ehrenberg-1 [40]	2023	F	5	2 Y	No change
Ehrenberg-10 [40]	2023	M	3	2 Y	No change
Ding [41]	2019	F	30	15 MO	No change

Year: Date of publication, F: Female, M: Male, F/U: Duration of reported follow-up, Y: Year, MO: Months

^a^Number following author name represents case number in the cited publication

^b^Age at initial presentation

## Vision loss secondary to TM

While TM is generally considered an asymptomatic and benign condition, vision loss may occur either because the torpedo lesion involves the fovea or secondary to complications arising from the torpedo lesion, such as choroidal neovascularization.

In addition to Case 2 reported herein, there are few other reported cases of torpedo lesions that involve the fovea. Makino and Tampo report on an unusual case of TM in the right eye of an 8 year old boy [18]. The torpedo lesion in the temporal macula had a satellite lesion superior to the main lesion and together they distorted the macula resulting in 0.03 (20/666) visual acuity. OCT through the lesion revealed inner retinal hyper-reflectivity with reduced thickness and disruption of the photoreceptor and retinal pigment epithelium layers. Giannakaki-Zimmermann, et al. reported on a patient with a fovea-involving torpedo lesion in the right eye resulting in 20/50 visual acuity [19]. They found that the torpedo lesion caused focal loss of the ellipsoid zone and interdigitation zone within the foveola. Light and Liu reported on a patient with a multi-partite torpedo lesion that was centered on the fovea of the left eye and resulted in a visual acuity of 20/40 [6]. OCT scan through the fovea of this eye also showed loss of the ellipsoid zone. Thus, if the torpedo lesion involves the fovea the patient’s visual acuity will be reduced.

The most frequently reported vision-threatening complication of TM is choroidal neovascularization. This appears to be a relatively uncommon complication, as our review of the literature revealed only 8 reported cases [4, 12, 20–25]. In six of the 8 cases (75%), the involved torpedo lesions contained a choroidal excavation. Because choroidal excavation is known to be associated with choroidal neovascularization [26], the presence of CNV in these eyes may be a consequence of choroidal excavation rather than a complication of TM itself. However, it is interesting to note that the 2 reported cases of CNV occurring in torpedo lesions without choroidal excavation are the youngest patients reported with this complication. The mean age of the two reported cases of CNV affecting torpedo lesions without choroidal excavation is 14.5 years compared to a mean age of 41 years for the 6 cases with choroidal excavation.

## Differential diagnosis

Because the diagnosis of TM is made based on the clinical features of the condition, it is necessary to consider a variety of other retinal lesions that may share some features with TM before a diagnosis can be made. Conditions included in the differential diagnosis of TM are chorioretinal scar, congenital hypertrophy of the RPE, Gardner syndrome [27], and other syndromes associated with torpedo-like retinal lesions (such as Turcot syndrome [28], congenital Zika virus infection [29], and enhanced S-cone syndrome [30]).

Gardner syndrome is a variant of Familial Adenomatous Polyposis (FAP) where the retinal or other extraintestinal features are especially prominent. FAP is a condition caused by germline mutations in the adenomatous polyposis coli (APC) gene resulting in the development of hundreds to thousands of colorectal adenomas [31]. Left untreated approximately 100% of individuals with FAP will develop colorectal cancer. Approximately 70–80% of patients with FAP have pigmented retinal lesions, some of which have a torpedo-like shape [32].

It has recently been suggested that because Gardner syndrome and TM share numerous clinical features that they may share a common genetic etiology and pathogenesis [15]. Packo and Goldberg point out that the size, shape, variable pigmentation, adjacent depigmented halos and pigmented borders, presence of a tail, localized scotomas and subjacent choroidal excavation that characterize torpedo lesions in TM are all similar to the torpedo-like lesions of Gardner syndrome. Because of the pleiotropism and variable expressivity of Gardner syndrome, it is possible that TM represents a subclinical form of the systemic disorder. Therefore, Packo and Goldberg recommend that patients with TM (1) undergo evaluations for mutations in the APC gene, (2) undergo colonoscopy for intestinal polyps, and (3) individuals genetically related to patients with TM undergo inspection of their fundi for lesions characteristic of Gardner syndrome.

The clinical abnormalities associated with Gardner syndrome include supernumerary teeth, cranial osteomas, epidermal cysts, intestinal polyposis, brain and other tumors, and pigmented ocular fundus lesions [15]. Because our Case 1 reported a history of supernumerary teeth, we recommended genetic testing of the APC gene. Full gene sequencing with deletion and duplication analysis was undertaken. No pathogenic mutations, variants of unknown significance, or gross deletions or duplications were detected in the APC gene. Furthermore, a colonoscopy was ordered, and its results were normal. While we were unable to examine family members ourselves, we were informed that they all had normal eye examinations. Genetic testing was offered to Case 2 but it was declined.

Strengths of this case series are multimodal assessment of two patients with torpedo lesions at 5 and 10 years from baseline, which is, to our knowledge, the longest reported follow-up of the condition to date. Gene sequence and deletion/duplication analysis of the APC gene was performed on one patient with TM, which to our knowledge has not been previously reported. Limitations include examinations performed by various clinicians at multiple sites using differing instruments over the follow-up period which may adversely affect the ability to detect change.

## Conclusion

We report on the long-term follow-up of two cases of TM. We found no clinically significant progression of the disorder over a 10 year observation period. Lesion morphology and visual function remained largely stable in both patients, with only minor degenerative changes observed in one case. Furthermore, genetic testing and colonoscopy of one of our patients found no evidence of Gardner syndrome. Our findings support the position that TM is an essentially stable, nonprogressive disorder. While we could find no evidence of Gardner syndrome in our patient, we endorse the recommendation that individuals with TM and possible manifestations of Gardner syndrome should undergo investigations to rule out the condition.

## Data Availability

Data sharing is not applicable to this article as no datasets were generated or analyzed during the current study.
